# Supplementary material for: Coordination between Surface Lattice Resonances of Poly(glycidyl Methacrylate) Line Array and Surface Plasmon Resonances of CdS Quantum on Silicon Surface
Source: Polymers (Basel). 2019 Mar 25;11(3):558. doi: 10.3390/polym11030558 (PMC6473753; doi:10.3390/polym11030558)
Supplement: Supplementary file 1 [file polymers-11-00558-s001.pdf]

polymers

MDPI

Figure S1: (a) XPS survey spectrum of a surface presenting pristine silicon, Si-AS, Si-AS-BB, Si-PGMA and Si-PGMA-CQD. (b) XPS C 1s core level spectrum of the Si-PGMA-CQD. XPS (c) Cd 3d and (d) S 2p core level spectra of the Si-PGMA-CQD functionalized surface.

(a)

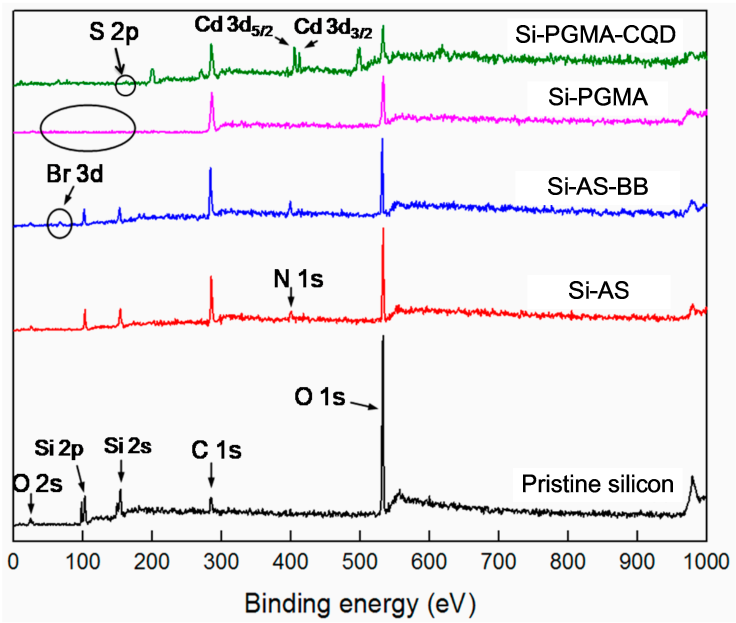

(b)

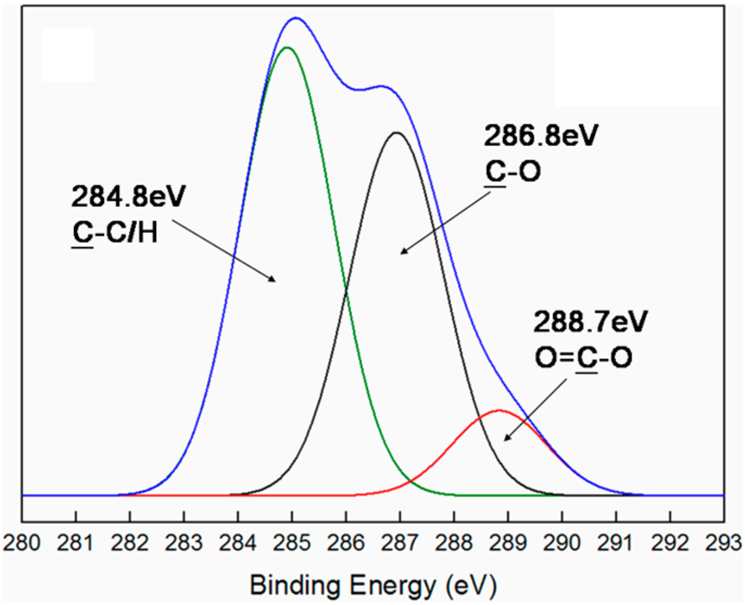

(c)

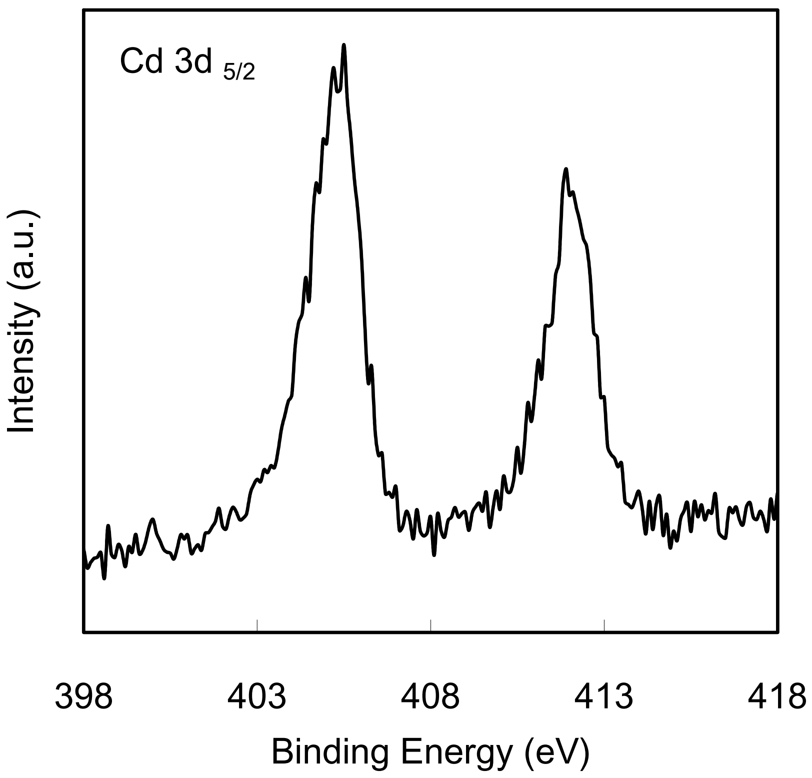
Cd 3d 5/2
Intensity (a.u.)
398
403
408
413
418
Binding Energy (eV)

(d)

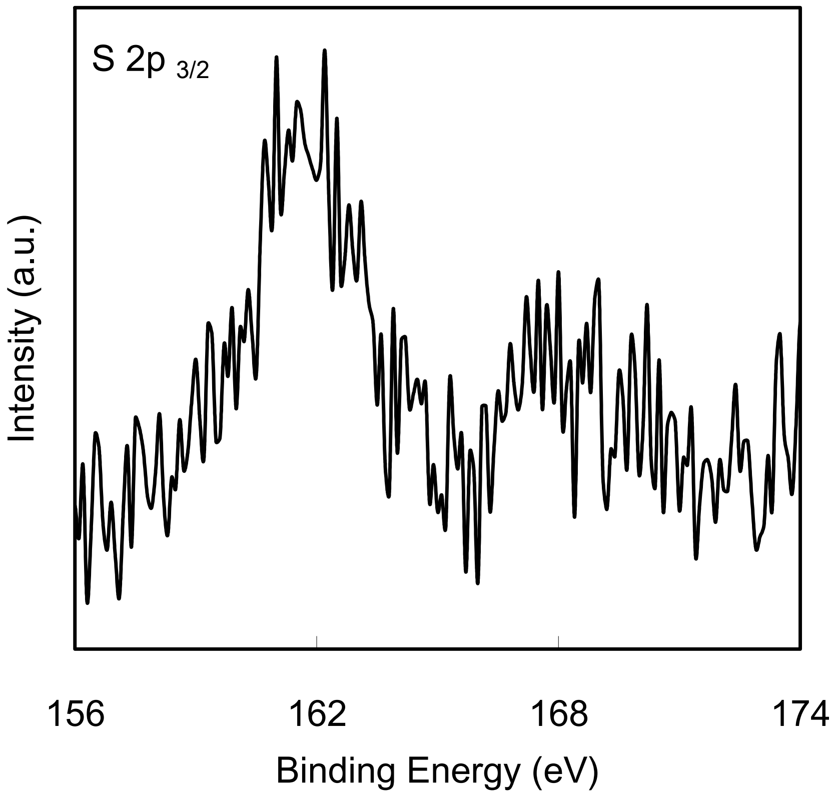
S 2p 3/2
Intensity (a.u.)
156
162
168
174
Binding Energy (eV)
